# Supplementary material for: Genetic regulation of OAS1 nonsense-mediated decay underlies association with COVID-19 hospitalization in patients of European and African ancestries
Source: Nat Genet. 2022 Jul 14;54(8):1103–16. doi: 10.1038/s41588-022-01113-z (PMC9355882; doi:10.1038/s41588-022-01113-z)
Supplement: Supplementary file 1 — Supplementary Figures 1–5 and Source Data for Supplementary Figure 3. [file 41588_2022_1113_MOESM1_ESM.pdf]

nature portfolio

https://doi.org/10.1038/s41588-022-01113-z

Supplementary information

Genetic regulation of *OAS1* nonsense-mediated decay underlies association with COVID-19 hospitalization in patients of European and African ancestries

In the format provided by the authors and unedited

Supplementary Materials

Genetic regulation of *OAS1* nonsense-mediated decay underlies association with COVID-19 severity in patients of European and African ancestries

Banday*, Stanifer*, Florez-Vargas* et al

Supplementary Figures

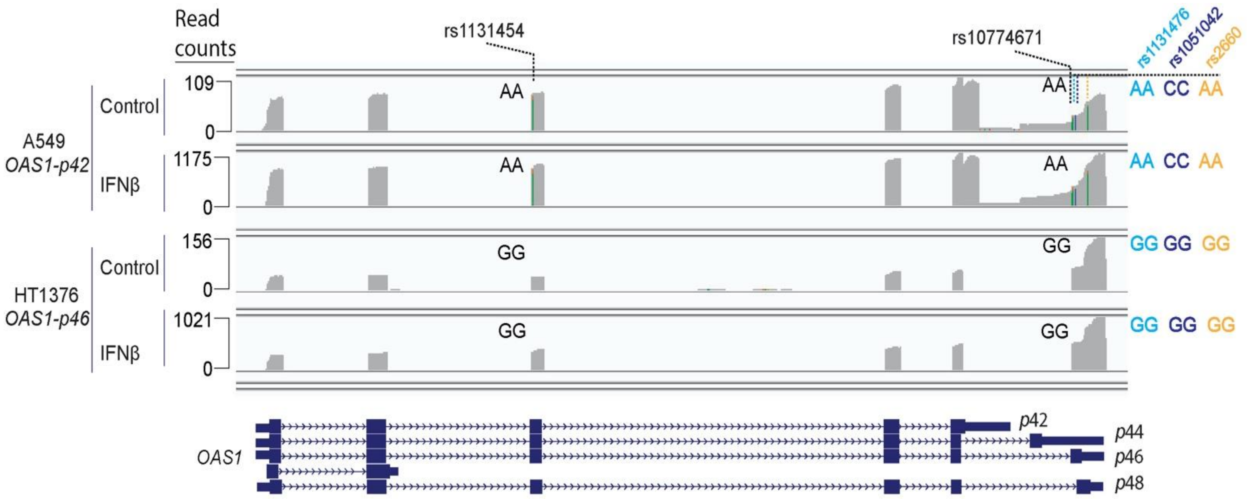

Supplementary Fig. 1. Long-read sequencing of *OAS1* transcripts in A549 and HT1376 cells with and without IFNβ treatment.

The A549 (rs10774671-AA, *OAS1-p42*) and HT1376 (rs10774671-GG, *OAS1-p46*) cells were treated with IFNβ or PBS (control) for 48 hrs. Total RNA was harvested and subjected to long-read sequencing with Oxford Nanopore. In both cell lines, the expression of *OAS1* isoforms was strongly induced by IFNβ treatment. The IGV plots show auto-scaled profiles with read counts indicating maximum coverage in the window. Genotypes of transcribed variants are indicated. Source: NCBI SRA: PRJNA743928, this paper.

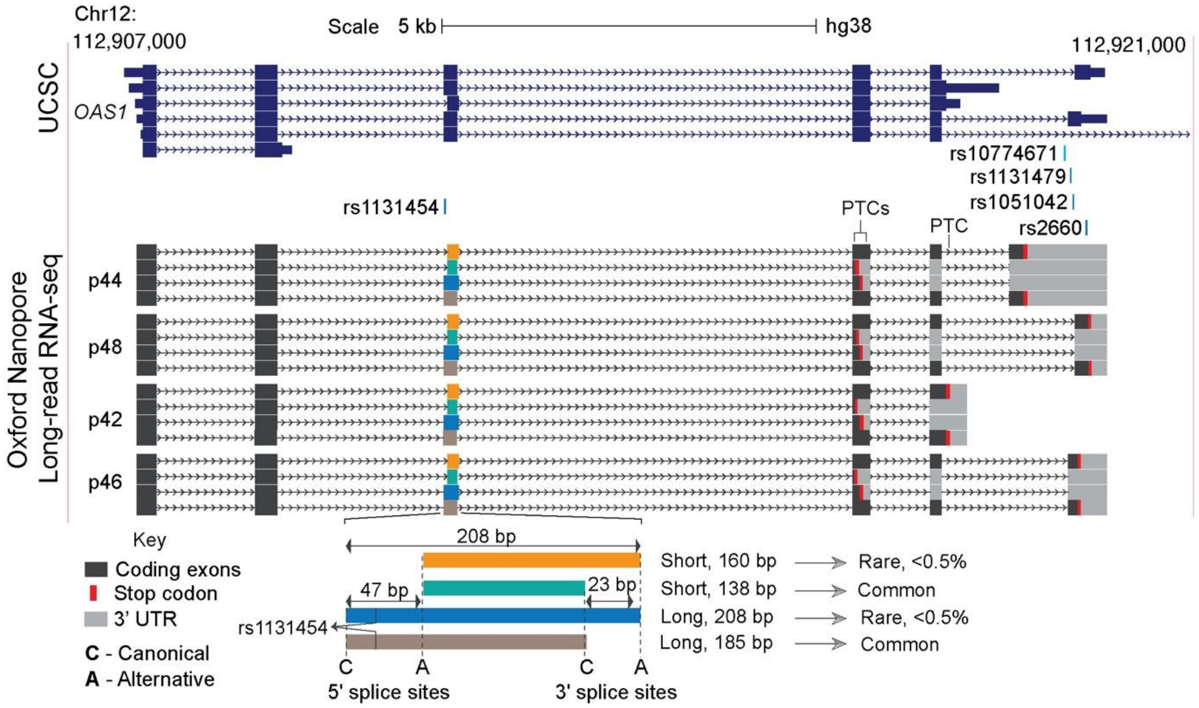

Supplementary Fig. 2. Long-read sequencing of *OAS1* transcripts demonstrates functional consequences of alternative splicing of exon 3.

UCSC genome browser view shows the known *OAS1* transcripts and full-length sequences of *OAS1* isoforms determined by long-read RNA-seq with Oxford Nanopore (**Supplementary Fig. 1**). Splicing patterns of *OAS1* exon 3 depends on two acceptor and two donor splice sites that define the Short and Long exon 3 isoforms. Two common exon 3 isoforms are Short (138 bp) and Long (185bp), while rare (<5% reads) exon 3 isoforms are Short (160 bp) and Long (208 bp). The inclusion of common Short (138 bp) or rare Long (208 bp) exon 3 isoforms results in premature termination codons (PTC) in exon 4. The stop codon of canonical *p42* isoforms presented by rare Short (160 bp) and common Long (185 bp) isoforms is located within exon 5 and functions as a PTC due to the inclusion of additional exons creating *p44* or *p48* isoforms. All transcripts with PTC are targeted by nonsense-mediated decay (NMD) with a variable extent of degradation depending on specific isoforms. Source: NCBI SRA: PRJNA743928, this paper.

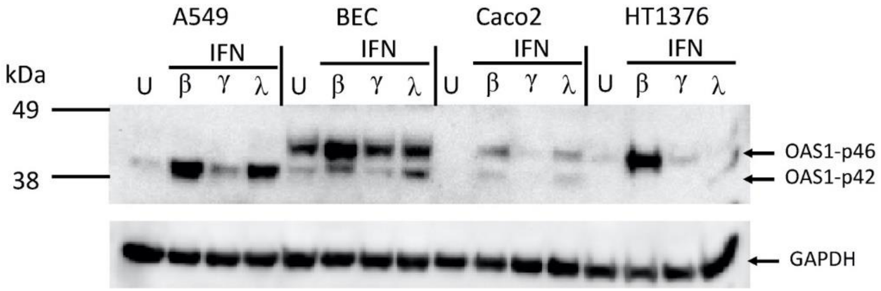

Supplementary Fig. 3. Western blot analysis of endogenous OAS1-p42 and p46 protein isoforms in human cell lines.

Four cell lines with different genotypes of rs10774671: A549 (AA), primary bronchial epithelial cells BEC (AG), Caco2 (AG), and HT1376 (GG) were untreated (U) or treated with interferons – IFNβ (2ng/mL), IFNγ (2ng/mL) or IFN-λ3 (100 ng/mL) for 48 hrs. Similar amounts of protein lysates were used for western blot with an antibody for the C-terminal part of OAS1. Only two isoforms, OAS1-p42 and p46, were detected, with the p46 protein isoform being dominant in heterozygous cell lines. In homozygous cell lines, the protein expression of p42 and p46 appeared comparable after IFN treatment. The experiment was independently repeated 3 times with comparable results; the results of one experiment are presented. Unprocessed blots are shown in **Source Data for Supplementary Fig. 3.**

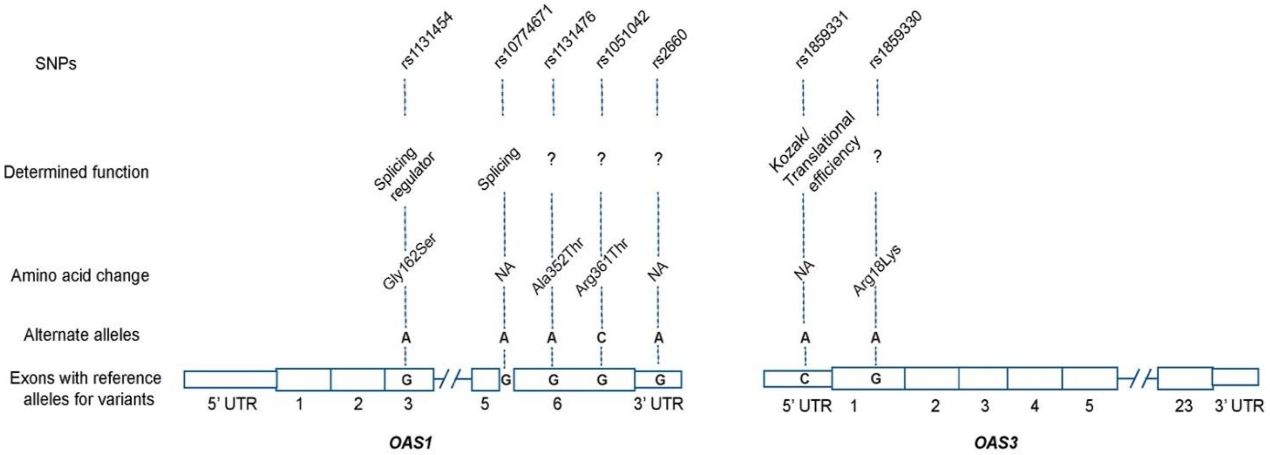

Supplementary Fig. 4. Potentially functional exonic variants within *OAS1* and *OAS3*.

Annotation of *OAS1* and *OAS3* exonic variants associated with COVID-19 disease severity in patients of European ancestry (P=6.4E-04 to 2.88E-03) and with suggestive associations in patients of African ancestry (P=2.05E-02 to 9.26E-02, **Supplementary Table 1a**).

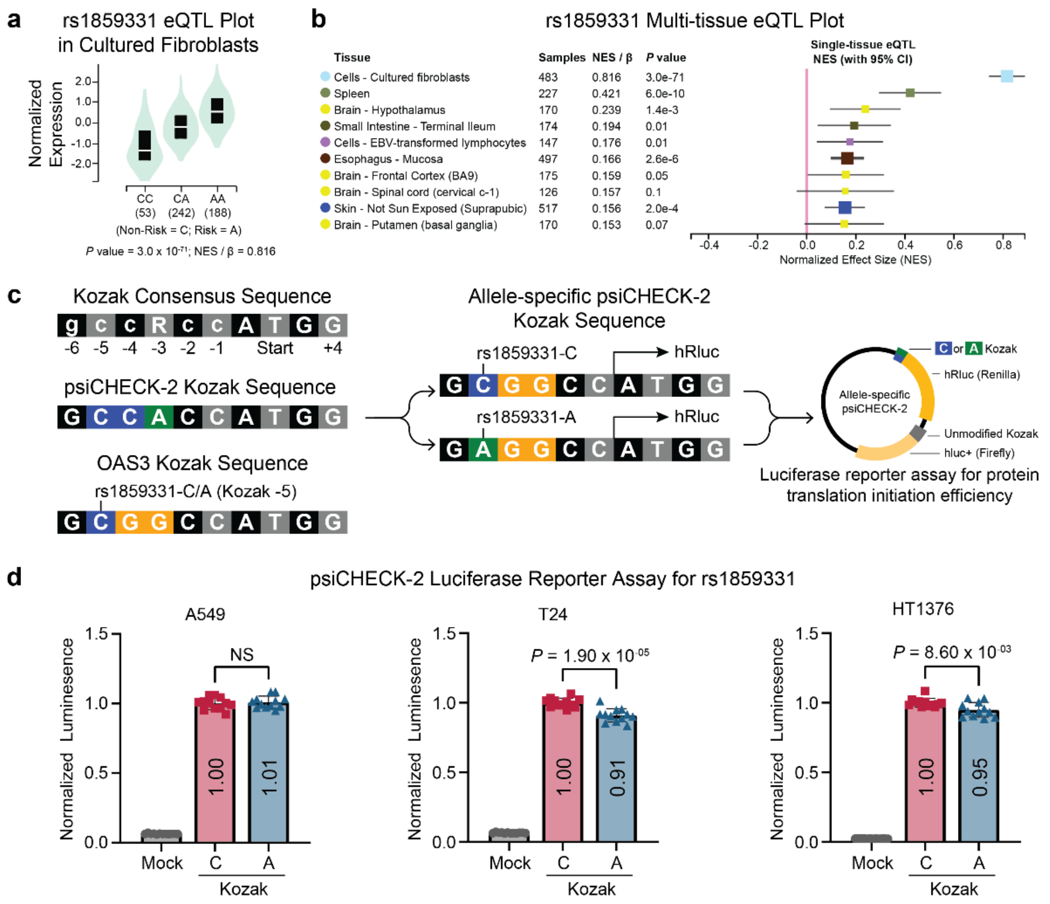

Supplementary Fig. 5. Functional effects of rs1859331 within the 5'UTR of *OAS3*.

a, eQTL plot for *OAS3* expression in cultured fibroblasts (n=483) in the Genotype-Tissue Expression (GTEx) dataset in relation to *OAS3*-5'UTR rs1859331 (non-risk allele = C, risk allele = A). Boxplots show the median values with 25th and 75th percentile distribution. **b,** A multi-tissue eQTL plot for *OAS3*-5'UTR-rs1859331 in a subset of tissue types in GTEx. Per-allele normalized effect sizes (NES) are plotted as beta (β) coefficients with 95% confidence intervals (CIs). *P*-values from GTEx eQTL are for linear regression models of gene expression and genotypes. **c,** Comparison of Kozak protein translation initiation sequences – consensus, Renilla (hRLuc) of psiCHECK-2 plasmid, and *OAS3*. The Kozak sequence of hRLuc of psiCHECK-2 was modified through site-directed mutagenesis at a -5bp position upstream of the translation start site (ATG) to match allelic forms of rs1859331 within the Kozak sequence of *OAS3*. **d,** Allele-specific Luciferase reporter assays for protein initiation efficiency modulated by Kozak sequences in A549, T24, and HT1376 cell lines. Cells were mock-transfected (no plasmids) or transfected with rs1859331 allele-specific psiCHECK-2 plasmids, all 12 biological replicates per condition; the results are presented mock-normalized with means and s.d.; *P*-values are for unpaired, two-sided Student's *t*-tests, NS – not significant. The rs1859331-A risk allele is associated with increased *OAS3* mRNA expression but decreased protein translation initiation efficiency in an *in vitro* model in some cell lines (T24 and HT1376) but not A549.

Source Data for Supplementary Fig. 3. Unprocessed western blots

Anti-OAS1antibody

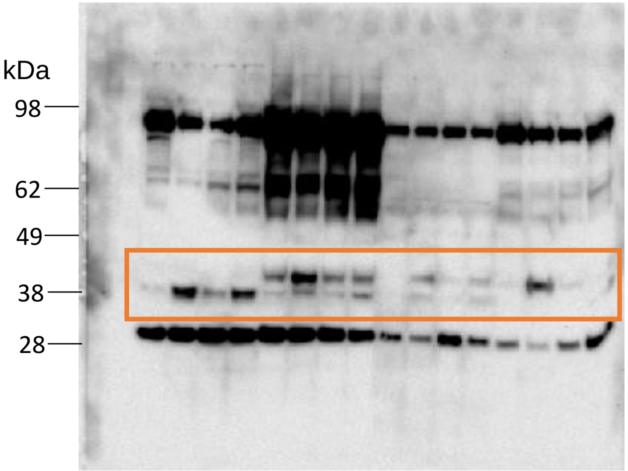

Anti-GAPDH antibody

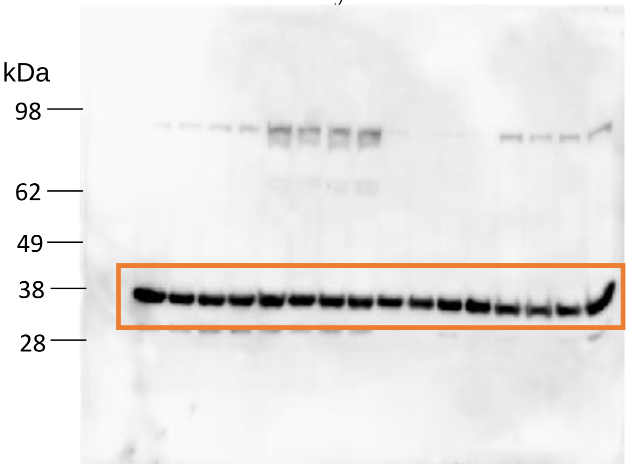

Supplementary References

1. Jin, Y., Schaffer, A.A., Feolo, M., Holmes, J.B. & Kattman, B.L. GRAF-pop: A Fast Distance-Based Method To Infer Subject Ancestry from Multiple Genotype Datasets Without Principal Components Analysis. *G3 (Bethesda)* **9**, 2447-2461 (2019).
2. Taliun, D. *et al.* Sequencing of 53,831 diverse genomes from the NHLBI TOPMed Program. *Nature* **590**, 290-299 (2021).
3. R Core Team. R: A language and environment for statistical computing. (R Foundation for Statistical Computing, Vienna, Austria, 2014).
4. Delaneau, O., Marchini, J. & Zagury, J.F. A linear complexity phasing method for thousands of genomes. *Nat Methods* **9**, 179-81 (2011).
5. Purcell, S. *et al.* PLINK: a tool set for whole-genome association and population-based linkage analyses. *Am J Hum Genet* **81**, 559-75 (2007).
6. Yang, J., Lee, S.H., Goddard, M.E. & Visscher, P.M. GCTA: a tool for genome-wide complex trait analysis. *Am J Hum Genet* **88**, 76-82 (2011).
7. Manichaikul, A. *et al.* Robust relationship inference in genome-wide association studies. *Bioinformatics* **26**, 2867-73 (2010).
8. Ferguson, W., Dvora, S., Fikes, R.W., Stone, A.C. & Boissinot, S. Long-term balancing selection at the antiviral gene OAS1 in Central African chimpanzees. *Mol Biol Evol* **29**, 1093-103 (2012).
