# Supplementary material for: Genetic regulation of OAS1 nonsense-mediated decay underlies association with COVID-19 hospitalization in patients of European and African ancestries
Source: Nat Genet. 2022 Jul 14;54(8):1103–16. doi: 10.1038/s41588-022-01113-z (PMC9355882; doi:10.1038/s41588-022-01113-z)
Supplement: Supplementary file 2 — Reporting Summary [file 41588_2022_1113_MOESM2_ESM.pdf]

nature research

Corresponding author(s): Prokunina-Olsson

Last updated by author(s): 05/10/2022

Reporting Summary

Nature Research wishes to improve the reproducibility of the work that we publish. This form provides structure for consistency and transparency in reporting. For further information on Nature Research policies, see our Editorial Policies and the Editorial Policy Checklist.

Statistics

For all statistical analyses, confirm that the following items are present in the figure legend, table legend, main text, or Methods section.

n/a	Confirmed	
☐	☒	The exact sample size (*n*) for each experimental group/condition, given as a discrete number and unit of measurement
☐	☒	A statement on whether measurements were taken from distinct samples or whether the same sample was measured repeatedly
☐	☒	The statistical test(s) used AND whether they are one- or two-sided<br>*Only common tests should be described solely by name; describe more complex techniques in the Methods section.*
☐	☒	A description of all covariates tested
☐	☒	A description of any assumptions or corrections, such as tests of normality and adjustment for multiple comparisons
☐	☒	A full description of the statistical parameters including central tendency (e.g. means) or other basic estimates (e.g. regression coefficient) AND variation (e.g. standard deviation) or associated estimates of uncertainty (e.g. confidence intervals)
☐	☒	For null hypothesis testing, the test statistic (e.g. *F*, *t*, *r*) with confidence intervals, effect sizes, degrees of freedom and *P* value noted<br>*Give P values as exact values whenever suitable.*
☒	☐	For Bayesian analysis, information on the choice of priors and Markov chain Monte Carlo settings
☐	☒	For hierarchical and complex designs, identification of the appropriate level for tests and full reporting of outcomes
☐	☒	Estimates of effect sizes (e.g. Cohen's *d*, Pearson's *r*), indicating how they were calculated

Our web collection on statistics for biologists contains articles on many of the points above.

Software and code

Policy information about availability of computer code

Data collection

1. Patients were recruited by several studies participating in COVNET: Large-scale Genome-wide Association Study and Whole Genome Sequencing of COVID-19 Severity (https://dceg.cancer.gov/research/how-we-study/genomic-studies/covnet).
2. COVNET DNA samples were analyzed by AmpFLSTR Identifiler and then genotyped for 712,191 variants using the Global Screening Array version 2.0 (GSA2, Illumina)
3. RNA-seq, ATAC-seq, H3K27ac ChIP-seq, and Hi-C datasets used in this study are listed in Table S15. These data were downloaded from NCBI SRA using SRA toolkit version 2.3.2.

Data analysis

1. CONVET genetic data quality control was performed with PLINK (v1.9) and KING (v2.2.7). Ancestry estimation was done with GRAF (v2.3.1). Principal components analysis (PCA) of genetic data was performed with GCTA (v1.93.0 beta) to generate the first 20 eigenvectors, separately for each ancestry.
2. Imputation variants was performed using the TOPMed imputation server (https://imputation.biodatacatalyst.nhlbi.nih.gov/#!). Logistic regression analysis of genetic data with phenotypes were done with using glm function in R (v4.0.4). For haplotype analyses, ShapeIT (v2.r837) software was used to phase the selected variants and PLINK (v1.07) was used to perform associations. LD plots were generated with Haploview (v4.2).
3. The FASTQ files were compressed using GZIP (version 1.10) and aligned with STAR version 2.7.6a to the GRChg38/hg38 or hg19 genome assembly.
4. Bam files were indexed and sliced for specific genomic regions using SAM tools version 1.11
5. Exon quantification from BAM files was performed using ASpli package (version 1.5.1: https://bioconductor.org/packages/release/bioc/vignettes/ASpli/inst/doc/ASpli.pdf) on R platform (versions 3.6.0 to 4.0.4).
6. For ATAC-seq and H3K27ac ChIP-seq analysis, the FASTQ files were aligned to hg19 using ENCODE-DCC ATAC-seq-pipeline version 1.9.1 (https://github.com/ENCODE-DCC/atac-seq-pipeline) and ChiP-seq-pipeline2 version 1.6.1 (https://github.com/ENCODE-DCC/chip-seq-pipeline2) with default settings. The output bigwig files were then uploaded to the UCSC genome browser for visualization.
7. Hi-C, FASTQ files were processed using Juicer 1.6 (https://github.com/aidenlab/juicer) by selecting relevant restriction cutting sites such as Mbo I/Dpn II and aligned to hg19. The chromatin loops in Hi-C data were detected using Hiccups in Juicer tools with default settings.

8. Integrative Genomics Viewer version 2.8.9 (http://www.broadinstitute.org/igv) was used for RNA-seq visualization
9. For identification of splicing factor binding motifs in exons, we used: Human Splicing Finder (HSF, www.umd.be/HSF3/)
10. Statistical analyses and plotting was performed using packages in R versions 3.6.0 to 4.0.4 and Prism - GraphPad (version 8)
11. Longitudinal trajectories of viral load in relation to genetic variants for clinical trail data were explored using a linear mixed-effects model function from the R nlme package (v3.1-153) to build linear mixed models
12. Nanopore GridION generated FASTQ files of long-read sequences were trimmed using Porechop version 0.2.4 and aligned to the hg19 genome using Minimap2 version 2.18

For manuscripts utilizing custom algorithms or software that are central to the research but not yet described in published literature, software must be made available to editors and reviewers. We strongly encourage code deposition in a community repository (e.g. GitHub). See the Nature Research guidelines for submitting code & software for further information.

Data

Policy information about availability of data

All manuscripts must include a data availability statement. This statement should provide the following information, where applicable:

- Accession codes, unique identifiers, or web links for publicly available datasets
- A list of figures that have associated raw data
- A description of any restrictions on data availability

Summary statistics for all genetic analyses is provided in Supplementary Tables. Dataset for Oxford Nanopore RNA-seq was deposited as SRA: PRJNA743928. Full-length sequence data for OAS1-p42 transcript with Short exon 3 was deposited to NCBI GenBank with accession number MZ491787. Requests for any additional data or reagents should be addressed to L.P.-O. (prokuninal@mail.nih.gov).

Field-specific reporting

Please select the one below that is the best fit for your research. If you are not sure, read the appropriate sections before making your selection.

☒ Life sciences ☐ Behavioural & social sciences ☐ Ecological, evolutionary & environmental sciences

For a reference copy of the document with all sections, see nature.com/documents/nr-reporting-summary-flat.pdf

Life sciences study design

All studies must disclose on these points even when the disclosure is negative.

Sample size	Sample size information for all types of data used in this study in provided in supplementary Tables. For omics data sets accessed from SRA, none of the samples were excluded from analysis based on 80% mappable sequence read cut-off. All samples with available data were used based on specific criteria such as genotypes.
Data exclusions	Except duplicates no samples were excluded from the analysis.
Replication	Yes, all experimental findings were reliably reproduced in multiple biological and technical replicates. Results were verified with minimum of three independent experiments.
Randomization	Not applicable for laboratory-based experimental data or genetic association studies
Blinding	All samples were processed blindly to their phenotype status

Reporting for specific materials, systems and methods

We require information from authors about some types of materials, experimental systems and methods used in many studies. Here, indicate whether each material, system or method listed is relevant to your study. If you are not sure if a list item applies to your research, read the appropriate section before selecting a response.

Materials & experimental systems

n/a	Involved in the study	
☐	☒	Antibodies
☐	☒	Eukaryotic cell lines
☒	☐	Palaeontology and archaeology
☒	☐	Animals and other organisms
☐	☒	Human research participants
☐	☒	Clinical data
☒	☐	Dual use research of concern

Methods

n/a	Involved in the study	
☒	☐	ChIP-seq
☒	☐	Flow cytometry
☒	☐	MRI-based neuroimaging

Antibodies

Antibodies used

Target gene | Cat. No. | Source | Target species | Host | Tag | Dilution
1. OAS1 PA5-82113 f Human Rabbit 1:200
2. GAPDH Ab9485 Abcam Human Rabbit 1:500
3. Golgin-97 A-21270 Thermo Fisher Tag Mouse 1:250
4. Anti-rabbit Alexa Flour 488 A21202 ThermoFisher 1:500
5. Anti-rabbit Alexa Fluor 680 A10043 ThermoFisher 1:500
6. Anti-Flag F7425-2MG Sigma Human Rabbit 1:1000
7. GAPDH 97166 Cell Signaling Technology Human mouse 1:1000
8. Anti-rabbit ab97051 Abcam 1:10,000
9. Anti-mouse ab6789 Abcam 1:10,000

Validation

We used antibodies that were validated as evidenced by Western Blot or Immunofluorescence images provided by vendors. Additionally, we used positive and negative controls to further assess the validity of all antibodies.
Vendor specific web-pages describing validation of antibodies are as:
1. OAS1 PA5-82113 | https://www.thermofisher.com/antibody/product/OAS1-Antibody-Polyclonal/PA5-82113
2. GAPDH Ab9485 | https://www.abcam.com/GAPDH-antibody-Loading-Control-ab9485.html?gclsrc=aw.ds|aw.ds&gclid=CjwKCAjwve2TBhByEiwAaktM1BwiNq_fLMM0PVqI4Jvkzz3hieNwi4BJp1UPN53zcBRvKbDAwADpTxoC2yEQAvD_BwE
3. Golgin-97 A-21270 | https://www.thermofisher.com/antibody/product/Golgin-97-Antibody-clone-CDF4-Monoclonal/A-21270
4. Anti-rabbit Alexa Flour 488 A21202 | https://www.thermofisher.com/order/genome-database/generatePdf?productName=Mouse%20IgG%20(H+L)&assayType=PRANT&productId=A-21202&detailed=true
5. Anti-rabbit Alexa Fluor 680 A10043 | https://www.thermofisher.com/antibody/product/Donkey-anti-Rabbit-IgG-H-L-Highly-Cross-Adsorbed-Secondary-Antibody-Polyclonal/A10043
6. Anti-Flag F7425-2MG | https://www.sigmaaldrich.com/US/en/product/sigma/f7425
7. GAPDH 97166 | https://www.cellsignal.com/products/primary-antibodies/gapdh-d4c6r-mouse-mab/97166
8. Anti-rabbit ab97051 | https://www.abcam.com/goat-rabbit-igg-hl-hrp-ab97051.html
9. Anti-mouse ab6789 Abcam | https://www.abcam.com/goat-mouse-igg-hl-hrp-ab6789.html

Eukaryotic cell lines

Policy information about cell lines

Cell line source(s)

Most of the cell lines used were purchased from the American Type Culture Collection (ATCC). Detailed information is provided in Table S4.

Authentication

Cells lines were freshly purchased from ATCC or if used longer than 6 months, authenticated by genotyping of a panel of microsatellite markers - Identifiler, performed by the Cancer Genomics Research Laboratory, NCI
Cells Source
HT-1376 - ATCC ,STR profiling
Caco-2, ATCC ,used within 6 months of purchase
A549 ATCC STR profiling
ACE2-A549 stable - gift from Dr. Ralf Bartenschlager STR profiling
THP-1 - ATCC
T24-ATCC
SCaBER - ATCC
SW780- ATCC
HEBC - ATCC
Vero E6 - ATCC

Mycoplasma contamination

All cell lines in the laboratory are regularly tested for mycoplasma contamination using the MycoAlert Mycoplasma Detection kit (Lonza). Cell lines tested negative when compared to positive control

Commonly misidentified lines
(See ICLAC register)

No commonly misidentified cell lines were used.

Human research participants

Policy information about studies involving human research participants

Population characteristics

Describe the covariate-relevant population characteristics of the human research participants (e.g. age, gender, genotypic information, past and current diagnosis and treatment categories). If you filled out the behavioural & social sciences study design questions and have nothing to add here, write "See above."

Recruitment

Describe how participants were recruited. Outline any potential self-selection bias or other biases that may be present and how these are likely to impact results.

Ethics oversight | *Identify the organization(s) that approved the study protocol.*

Note that full information on the approval of the study protocol must also be provided in the manuscript.

Clinical data

Policy information about clinical studies

All manuscripts should comply with the ICMJE guidelines for publication of clinical research and a completed CONSORT checklist must be included with all submissions.

Clinical trial registration	We used published data and samples from a clinical trial NCT04354259
Study protocol	*Note where the full trial protocol can be accessed OR if not available, explain why.*
Data collection	*Describe the settings and locales of data collection, noting the time periods of recruitment and data collection.*
Outcomes	*Describe how you pre-defined primary and secondary outcome measures and how you assessed these measures.*
