# Supplementary figures and images for: Genetic regulation of OAS1 nonsense-mediated decay underlies association with COVID-19 hospitalization in patients of European and African ancestries
Source: Nat Genet. 2022 Jul 14;54(8):1103–16. doi: 10.1038/s41588-022-01113-z (PMC9355882; doi:10.1038/s41588-022-01113-z)

**Fig. 2c. Unprocessed western blots**  
Anti-OAS1 antibody

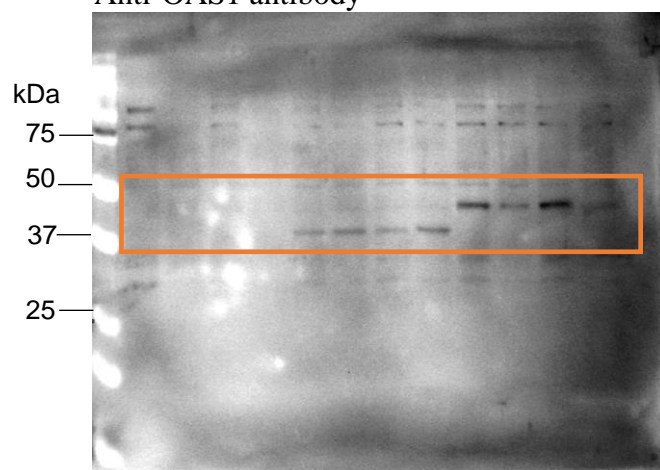

Anti-GAPDH antibody

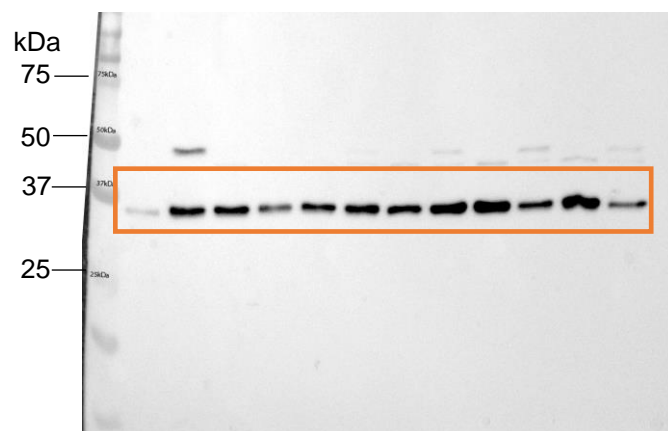

Supplement: Source Data Fig. 2 — Unprocessed western blots for Fig. 2c. [file 41588_2022_1113_MOESM5_ESM.pdf]

**Fig. 5c.** Unprocessed agarose gel image

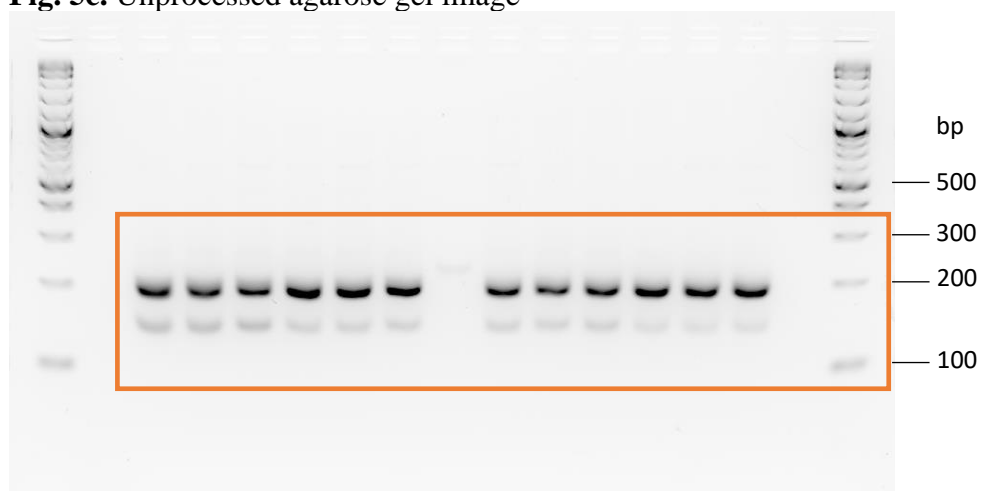

Supplement: Source Data Fig. 5 — Unprocessed agarose gel image for Fig. 5c. [file 41588_2022_1113_MOESM6_ESM.pdf]
